# Completeness of case ascertainment and survival time error in English cancer registries: impact on 1-year survival estimates

**DOI:** 10.1038/bjc.2011.168

**Published:** 2011-05-10

**Authors:** H Møller, S Richards, N Hanchett, S P Riaz, M Lüchtenborg, L Holmberg, D Robinson

**Affiliations:** 1 King's College London, Thames Cancer Registry, 42 Weston Street, London SE1 3QD, UK; 2 Cancer Epidemiology Unit, King's College London, Bermondsey Wing, Guy's Hospital, London SE1 9RT, UK

**Keywords:** survival, cancer registries, incompleteness

## Abstract

**Background:**

It has been suggested that cancer registries in England are too dependent on processing of information from death certificates, and consequently that cancer survival statistics reported for England are systematically biased and too low.

**Methods:**

We have linked routine cancer registration records for colorectal, lung, and breast cancer patients with information from the Hospital Episode Statistics (HES) database for the period 2001–2007. Based on record linkage with the HES database, records missing in the cancer register were identified, and dates of diagnosis were revised. The effects of those revisions on the estimated survival time and proportion of patients surviving for 1 year or more were studied. Cases that were absent in the cancer register and present in the HES data with a relevant diagnosis code and a relevant surgery code were used to estimate (a) the completeness of the cancer register. Differences in survival times calculated from the two data sources were used to estimate (b) the possible extent of error in the recorded survival time in the cancer register. Finally, we combined (a) and (b) to estimate (c) the resulting differences in 1-year cumulative survival estimates.

**Results:**

Completeness of case ascertainment in English cancer registries is high, around 98–99%. Using HES data added 1.9%, 0.4% and 2.0% to the number of colorectal, lung, and breast cancer registrations, respectively. Around 5–6% of rapidly fatal cancer registrations had survival time extended by more than a month, and almost 3% of rapidly fatal breast cancer records were extended by more than a year. The resulting impact on estimates of 1-year survival was small, amounting to 1.0, 0.8, and 0.4 percentage points for colorectal, lung, and breast cancer, respectively.

**Interpretation:**

English cancer registration data cannot be dismissed as unfit for the purpose of cancer survival analysis. However, investigators should retain a critical attitude to data quality and sources of error in international cancer survival studies.

It has been suggested that cancer registries in England are too dependent on the processing of information from death certificates (Bullard *et al*, 2000; Robinson *et al*, 2007; Beral and Peto, 2010; Møller *et al*, 2010). This could have several adverse effects: (a) case ascertainment would be incomplete, particularly for non-fatal cases; (b) survival time would be too short, because hospital activity related to recurrent disease or end-of-life care would sometimes be recorded as the first known event and hence provide the date of diagnosis. A further consequence of these errors would be that (c) reported cancer survival statistics for England would be estimated with a systematic bias and be too low.

Cancer registries in England have recently linked routine cancer registration records with information from the Hospital Episode Statistics (HES) database (http://www.hesonline.nhs.uk). The HES database contains details of all in-patient and day-case admissions to National Health Service (NHS) hospitals in England.

The linked data set provides a new opportunity to evaluate the magnitude of errors (a), (b), and (c), defined above. Based on record linkage with the HES database, records missing in the cancer register were identified, and dates of diagnosis were revised. The effects of those revisions on the estimated survival time and proportion of patients surviving for 1 year or more were studied. Cases that were absent in the cancer register and present in the HES data with a relevant diagnosis code and a relevant surgery code were used to estimate (a) the completeness of the cancer register. Differences in survival times calculated from the two data sources were used to estimate (b) the possible extent of error in the recorded survival time in the cancer register. Finally, we combined (a) and (b) to estimate (c) the resulting differences in 1-year cumulative survival estimates.

The analyses presented here were executed at the Thames Cancer Registry on behalf of cancer registries in England, in order to address the comments in a recent editorial by Beral and Peto (2010). The principal analysis and the form of reporting of findings were specified before we had any knowledge of the results of the investigation.

## Materials and methods

We identified all cancer records in the HES-only side of the linked data set that in 2001–2007 had activity relating to colorectal cancer (ICD10 C18–C21), lung cancer (C33–C34), or breast cancer (C50), and that had a surgery code indicating a relevant, non-diagnostic surgical procedure. By HES-only cases, we are referring to the cases identified by the record linkage, that are not present in the cancer register but present in the HES data with a relevant diagnosis code and a relevant surgery code. These cases would not be included in routine cancer survival analysis, and they represent good-prognosis cases that may have been missed in the primary case ascertainment in the cancer registries, and not subsequently identified through routine record linkage with death certificates.

The three cancer diagnosis groups were selected to represent the spectrum of fatality among different, common types of cancer. The HES-only records were considered as an indication of the possible magnitude of under-ascertainment of non-fatal cancer cases in the cancer registries.

We considered only surgically treated cases because the combination of diagnosis code and resection code in the HES record gives a high degree of certainty that the record represents a true record of cancer. The HES-only records without an indication of cancer treatment would not be considered as sufficient evidence to create a cancer registration, and would need to be verified against other clinical records. The large majority of such HES-only records are known to relate to cases where cancer might have been suspected but it was not subsequently confirmed (Brewster *et al*, 1997).

To give a measure of incompleteness, we compared the number of HES-only records (with surgical treatment) against the number of regular cancer registration records in the linked data set, and stratified this analysis by sex, age, year of diagnosis or HES activity (2001–2007), and cancer registry. We plotted the incompleteness measure for each cancer registry for each year.

To evaluate the possible magnitude of survival time error, we identified all cases in the cancer registry records with a recorded survival time of <1 year. These rapidly fatal cases were considered as the ones most likely to be influenced by a systematic survival time error. Within the three groups of cancers, we searched the HES records for evidence of an earlier cancer diagnosis for these persons (with or without a record of surgery), and used the first matching HES record with a relevant diagnosis code. We computed the difference in survival time (days) using the two alternative dates of diagnosis. We described the distributions of the survival time difference, stratified by type of cancer, and cancer registry.

Finally, we evaluated the likely magnitude of the influence of incompleteness and survival time error on a commonly used outcome measure: the 1-year survival proportion. We computed 1-year survival in three ways: (i) as routinely reported by cancer registries, (ii) with account taken of the HES-only cases and their respective 1-year survival, and (iii) with further account taken of the extent of possible survival time error in the cancer registration records.

## Results

**Figure 1:**
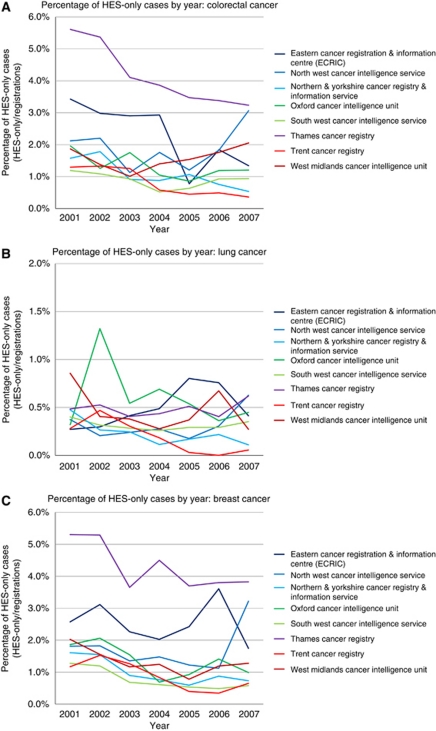
Percentage of HES-only cases in cancer registries in England, 2001–2007: (**A**) colorectal cancer, (**B**) lung cancer, and (**C**) breast cancer.

**Figure 2:**
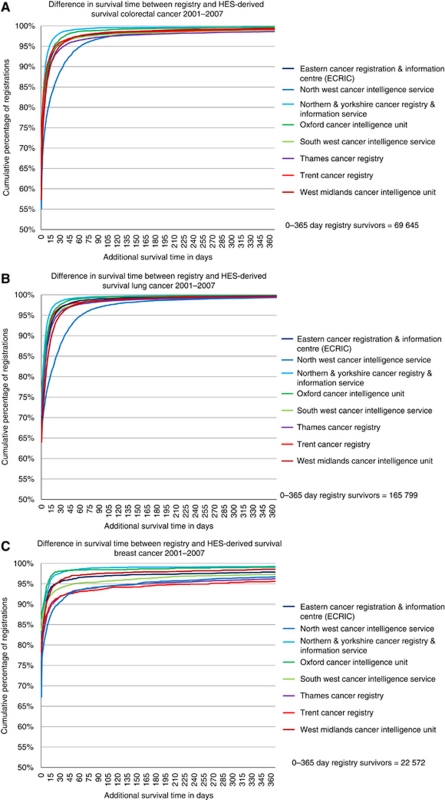
Cumulative distributions of difference in survival time according to cancer registration and HES records, England, 2001–2007: (**A**) colorectal cancer, (**B**) lung cancer, and (**C**) breast cancer.

Table 1 shows the estimated incompleteness of case ascertainment. The HES-only cases added 1.9% to the number of colorectal cancer registrations, 0.4% to lung cancer, and 2.0% to breast cancer. These effects were similar in males and females, slightly higher in the younger age groups, and declined over the period 2001–2007. There was some variation between cancer registries, with the highest incompleteness in the Thames Cancer Registry (4.1% in colorectal cancer, 0.5% in lung cancer, and 4.3% in breast cancer) and lowest in the Trent and the South West registries. There was a general decrease in incompleteness over time in most cancer registries (Figure 1). Table 2 and Figure 2 show the analysis of survival time error. The distribution of the difference between the two computed survival times (survival time according to HES data (HES-derived) *minus* survival time according to the cancer registry (registry-derived)) was extremely skewed. For colorectal cancer and lung cancer, 5.1% and 4.7% (respectively) of cases had a difference of >1 month, and 0.8% and 0.4% (respectively) had a difference of >1 year. Figures 2A and B show that the registries had similar cumulative distributions after about 3 months (i.e., the proportions of registrations with a difference of >3 months were similar across the registries). The North West cancer registry had a higher proportion of cases with a survival time difference of >1 month.

For breast cancer, 6.2% of cases had a survival time difference of more than 1 month and 2.7% differed by >1 year. There was variation in the distributions between the cancer registries, which persisted for >1 year (Figure 2C). The proportion of cases with survival difference of >1 year ranged from 0.8% in Northern and Yorkshire to 4.4% in Trent.

Table 3 shows the three alternative analyses of 1-year survival. The 1-year survival estimates increased when the HES-only cases and their respective survival times were considered in the analysis. The changes were small, amounting to 0.5, 0.2, and 0.2 percentage points for colorectal, lung, and breast cancer, respectively. With the further use of the HES-derived survival times for the cancer registration records, the 1-year survival times increased further but the changes remained small: 1.0, 0.8, and 0.4 percentage points, respectively.

## Discussion

The main findings from this analysis are that completeness of case ascertainment in English cancer registries is high, possibly as much as 98–99%, when evaluated against independently recorded hospital episodes which included relevant cancer diagnosis and surgery codes. The analysis found evidence of the hypothesised survival time error. Around 5–6% of rapidly fatal (1 year) cancer registrations had the survival time extended by more than a month and up to 3% of rapidly fatal breast cancer records were extended by more than a year. However, the resulting impact on estimates of 1-year survival was small, up to one percentage point for colorectal cancer. There was some variation in completeness and survival time difference between cancer registries.

There are important limitations of this analysis, and we do not propose that it gives a full and accurate estimation of completeness and survival time errors. The analysis uses a new source of data and a pre-specified analysis plan to indicate the possible magnitude and impact of the errors and biases proposed by Beral and Peto (2010) and previously investigated and discussed by ourselves (Bullard *et al*, 2000; Robinson *et al*, 2007; Møller *et al*, 2009, 2010). We decided *a priori* to consider only resected cases from HES as potentially missed cases in cancer registration, these would be representatives of the non-fatal cancers that the registration process might have missed (Bullard *et al*, 2000). The most likely reason for any absence of surgical treatment of hospitalised cancer patients would be that they were too ill to be considered eligible for surgery. The exclusion of such patients as potential cases is not likely to result in artificial underestimation of survival, but rather the contrary.

A small proportion of cancer patients may have had their diagnosis and surgery services provided in private hospitals, particularly patients residing in the London area. Such patients are not all recorded in the cancer registries and treatment services provided on a private basis are not recorded in HES.

The analysis suggests a slightly lower completeness in the youngest age groups, that is, colorectal and lung cancer patients below 50 years and breast cancer patients below 35 years (Table 1). This observation is based on small numbers. The good prognosis of young patients may be a contributing factor to this.

The principal limitation of the study lies in the completeness of the record linkage with the HES data and the accuracy of the information therein. Unique person identifiers (NHS numbers) have come to be almost universally used in NHS hospitals only in the last few years, and this puts a limit on the period covered in the linked data set. The year-on-year improvement in the availability of NHS numbers and in the completeness of the record linkage is the most likely reason for the slightly lower estimated completeness in 2001 and 2002 for colorectal cancer and breast cancer (Table 1).

Even in the most recent period, the linkage algorithm used NHS number, sex, date of birth, postcode, and date of death, and it is known to be imperfect. Some of the apparent HES-only cases will in fact have a corresponding record in the cancer registry, and there may be duplication whereby more than one of the HES-only cases relates to a single person.

Additionally, there are known errors in the routine HES data (as in any administrative data set) and some cases will have been missed because they did not include the specific cancer diagnosis or a relevant surgery code. We are not able to determine the direction and magnitude of errors created by these imperfections, but it seems unlikely that our analysis is severely flawed or biased. We will continue to explore means of quality assurance and improvement of the cancer registry records. The new linked data set will gradually improve through quality assurance processes related to the continuous use of the data and its annual updating.

Taken at face value, the 1–4% incompleteness in the Thames Cancer Registry is about as we would expect from previous analyses (Bullard *et al*, 2000; Robinson *et al*, 2007) and a recent update thereof (unpublished data, available on request). It is reassuring that most registries seem to have even higher completeness than Thames. The analysis of survival time differences between HES and cancer registries serves as a sensitivity analysis of survival estimates derived from English cancer registry data, but it should not be inferred that the earlier diagnosis date from HES is the correct one, particularly when the difference is small. The date of diagnosis concept in cancer registration does not always take the date of first hospital activity or first clinical diagnosis. In many cases, the date (often later) of the definitive histopathological diagnosis will prevail, in accordance with the international definition of date of diagnosis. The observed distribution of survival time differences in the North West cancer registry could be due to a more rigorous application of this rule, and does not necessarily point to a particular problem in the processing of death certificate information. The differences we have found between cancer registries will be explored by the registries and used in their continued quality assurance and improvement of the service.

In conclusion, we confirm the hypothesis (Beral and Peto, 2010) – and our own expectations (Bullard *et al*, 2000; Robinson *et al*, 2007; Møller *et al*, 2010) – that incompleteness of case ascertainment and survival time error are real phenomena which bias cancer survival estimates in the direction of too low estimates. The error is very small compared with the observed differences between North West European countries (Møller *et al*, 2009, 2010) and between socioeconomic groups in England (Møller *et al*, personal communication). Although the British situation, with immediate availability and processing of information from death certificates, entails a risk of dependence on this source of information, this is more desirable than the situation in several other European countries where death information can only be processed with technical difficulty and delay, or where it is considered as sensitive and not available for cancer registration (Møller *et al*, 2009). The estimates of completeness in cancer registries in England are generally consistent with estimates from other national cancer registries that process information from death certificates in the primary case ascertainment, for example, Finland (Robinson *et al*, 2007) and Norway (Larsen *et al*, 2009).

In the mid-1990s, the Thames Cancer Registry had 15–20% registrations based entirely on death certificates, and the data would not at present be considered as suitable for cancer survival analysis. This death certificate-only proportion has been gradually reduced to 1.6% in 2008. English cancer registration data can no longer be simply dismissed as unfit-for-purpose. It is worth noting that the errors we have discussed are not specific to the British situation but will exist in the same form or in similar forms in other countries as well. The best strategy is to be careful in the selection of comparison countries and to retain a critical (and self-critical) attitude to the international cancer survival and cancer mortality comparisons we perform (Holmberg *et al*, 2010; Møller *et al*, 2010; Morris *et al*, 2011).

## Figures and Tables

**Table 1:** Completeness of case ascertainment in cancer registries in England, 2001–2007, evaluated with HES records containing both a relevant diagnosis code and a relevant code for non-diagnostic surgery

	**Colorectal cancer**			**Lung cancer**			**Breast cancer**		
	**HESO**	**REPO**	**H/R (%)**	**HESO**	**REPO**	**H/R (%)**	**HESO**	**REPO**	**H/R (%)**
Total	4027	206 794	1.9	802	219 483	0.4	4921	251 201	2.0
									
*Sex*									
Male	2050	111 787	1.8	458	128 881	0.4	37	1797	2.1
Female	1948	95 007	2.1	344	90 602	0.4	4881	249 404	2.0
NA	29	0		0	0		3	0	
									
*Age (years)*									
0–4	0	3		0	7		0	1	
5–9	0	1		0	3		0	0	
10–14	4	21		0	8		1	1	
15–19	9	79		4	26		15	14	
20–24	15	249		3	37		17	125	
25–29	16	374		5	94		40	874	4.6
30–34	28	670	4.2	10	238		82	3317	2.5
35–39	48	1447	3.3	14	614		191	8398	2.3
40–44	80	2842	2.8	27	1722	1.6	290	15 257	1.9
45–49	117	4949	2.4	43	3992	1.1	416	20 787	2.0
50–54	172	8770	2.0	43	8660	0.5	509	29 098	1.7
55–59	302	15 613	1.9	111	16 463	0.7	587	31 710	1.9
60–64	398	20 368	2.0	119	23 762	0.5	625	30 317	2.1
65–69	512	26 795	1.9	117	30 799	0.4	583	27 210	2.1
70–74	643	32 600	2.0	153	38 454	0.4	484	22 559	2.1
75–79	698	35 483	2.0	111	41 973	0.3	502	22 167	2.3
80–84	553	30 667	1.8	33	31 891	0.1	332	19 093	1.7
85+	387	25 863	1.5	7	20 740	0.0	234	20 273	1.2
NA	45	0		2	0		13	0	
									
*Year*									
2001	751	28 329	2.7	139	31 141	0.4	865	34 352	2.5
2002	705	28 410	2.5	122	30 512	0.4	867	34 223	2.5
2003	546	28 787	1.9	101	30 757	0.3	637	36 261	1.8
2004	535	29 575	1.8	95	31 161	0.3	654	36 182	1.8
2005	435	30 167	1.4	107	31 684	0.3	567	36 990	1.5
2006	513	30 627	1.7	120	32 425	0.4	650	36 803	1.8
2007	542	30 899	1.8	118	31 803	0.4	681	36 390	1.9
									
*Registry*									
Eastern Cancer Registration & Information Centre (ECRIC)	527	23 232	2.3	106	21 396	0.5	722	28 501	2.5
North west Cancer Intelligence Service	533	27 881	1.9	111	35 384	0.3	562	32 831	1.7
Northern & Yorkshire Cancer Registry & Information Service	317	29 769	1.1	85	37 541	0.2	325	32 904	1.0
Oxford Cancer Intelligence Unit	134	10 220	1.3	54	9081	0.6	185	13 835	1.3
South west Cancer Intelligence Service	290	32 726	0.9	87	27 780	0.3	298	39 368	0.8
Thames Cancer Registry	1639	39 740	4.1	204	42 236	0.5	2224	51 915	4.3
Trent Cancer Registry	165	20 448	0.8	43	23 310	0.2	214	24 693	0.9
West Midlands Cancer Intelligence Unit	359	22 778	1.6	105	22 755	0.5	355	27 154	1.3
NA	63	0		7	0		36	0	

Abbreviations: HESO=Hospital Episode Statistics (HES)-only records from the linked repository (version 2007) with a surgery code for ‘major surgery’ H/R=HESO/REPO expressed as a percentage, computed for numerators >20 cases; NCIN=National Cancer Intelligence Network; REPO=valid cancer registrations from the linked repository. (These exclude the HESO records).

Analysis based on first occurrences of the particular type of cancer in a person.

Major surgery is defined as in the forthcoming NCIN surgery report. The definitions are available.

**Table 2:** Difference in survival time from date of diagnosis in cancer registration and from earliest episode in HES, England 2001–2007

	**Survival difference**				**Proportion that changed (%)**		
	**Same**	**Within 1 month**	**Within 1 year**	**Total**	**No change**	**More than 1 month**	**More than 1 year**
*Colorectal cancer registry*							
Eastern Cancer Registration & Information Centre (ECRIC)	4207	7134	7390	7460	56.4	4.4	0.9
North West Cancer Intelligence Service	5362	8652	9643	9729	55.1	11.1	0.9
Northern & Yorkshire Cancer Registry & Information Service	6366	9581	9730	9750	65.3	1.7	0.2
Oxford Cancer Intelligence Unit	2041	3142	3235	3244	62.9	3.1	0.3
South West Cancer Intelligence Service	7431	10 110	10 459	10 563	70.3	4.3	1.0
Thames Cancer Registry	8668	13 075	13 669	13 859	62.5	5.7	1.4
Trent cancer registry	4799	6993	7221	7284	65.9	4.0	0.9
West Midlands Cancer Intelligence Unit	4448	7374	7711	7756	57.3	4.9	0.6
Total	43 322	66 061	69 058	69 645	62.2	5.1	0.8
							
*Lung cancer registry*							
Eastern Cancer Registration & Information Centre (ECRIC)	10 627	15 534	16 004	16 045	66.2	3.2	0.3
North West Cancer Intelligence Service	17 740	23 860	26 910	27 094	65.5	11.9	0.7
Northern & Yorkshire Cancer Registry & Information Service	20 316	27 565	27 956	27 996	72.6	1.5	0.1
Oxford Cancer Intelligence Unit	5008	6769	6920	6931	72.3	2.3	0.2
South West Cancer Intelligence Service	15 843	20 318	20 978	21 079	75.2	3.6	0.5
Thames Cancer Registry	22 258	30 237	31 365	31 536	70.6	4.1	0.5
Trent Cancer Registry	13 096	17 106	17 554	17 634	74.3	3.0	0.5
West Midlands Cancer Intelligence Unit	11 193	16 652	17 430	17 484	64.0	4.8	0.3
Total	116 081	158 041	165 117	165 799	70.0	4.7	0.4
							
*Breast cancer registry*							
Eastern Cancer Registration & Information Centre (ECRIC)	1801	2183	2238	2287	78.7	4.5	2.1
North West Cancer Intelligence Service	2113	2847	3037	3141	67.3	9.4	3.3
Northern & Yorkshire Cancer Registry & Information Service	2002	2422	2462	2481	80.7	2.4	0.8
Oxford Cancer Intelligence Unit	900	1020	1030	1040	86.5	1.9	1.0
South West Cancer Intelligence Service	3194	3592	3719	3821	83.6	6.0	2.7
Thames Cancer Registry	3684	4383	4588	4768	77.3	8.1	3.8
Trent Cancer Registry	2112	2472	2569	2687	78.6	8.0	4.4
West Midlands Cancer Intelligence Unit	1838	2250	2313	2347	78.3	4.1	1.4
Total	17 644	21 169	21 956	22 572	78.2	6.2	2.7

All cases died within 1 year from diagnosis according to the cancer registry record.

**Table 3:** Case numbers and deaths within 1 year according to cancer registry data and HES data, and impact on one-year survival estimates, England, 2001–2007

	**Case numbers**					**One-year survival estimate (%)**		
**Cancer group**	**Registry cases diagnosed 2001–2007**	**HES-only cases diagnosed 2001–2007**	**Registry cases died within 365 days**	**HES-only cases died within 365 days**	**Registry cases died within 365 days using revised diagnosis date**	**According to registry records**	**According to registry and HES-only records**	**According to registry (using revised diagnosis date) and HES-only records**
	A	B	C	D	E	(1)	(2)	(3)
Colorectal cancer	206 794	4027	69 645	353	68 654	66.3	66.8	67.3
Lung cancer	219 483	802	165 799	46	164 485	24.5	24.7	25.3
Breast cancer	251 201	4921	22 572	82	21 858	91.0	91.2	91.4

Abbreviation: HES=Hospital Episode Statistics.

A: as reported in Table 1 on completeness of case ascertainment; B: as reported in Table 1 on completeness of case ascertainment; C: these cases died within 1 year according to the cancer registry data; D: these HES-only cases died within 1 year according to the HES data; E: with date of diagnosis revised, some registry cases now survive longer than 1 year.

(1)=(A–C)/A; (2)=((A+B)–(C+D))/(A+B); (3)=((A+B)–(E+D))/(A+B).
